# The Delayed Presentation of Abdominal Apoplexy Following Initial Negative Imaging: A Diagnostic Challenge

**DOI:** 10.7759/cureus.88156

**Published:** 2025-07-17

**Authors:** Wafa Iftekhar, Wei M Lim, Yeng Kwang Tay

**Affiliations:** 1 Department of Colorectal Surgery, Monash Health, Melbourne, AUS

**Keywords:** abdominal apoplexy, damage control laparotomy, idiopathic spontaneous intraperitoneal hemorrhage, intra-abdominal hemorrhage, middle colic artery rupture

## Abstract

Abdominal apoplexy, or idiopathic spontaneous intraperitoneal hemorrhage (ISIH), is a rare and life-threatening condition caused by the rupture of a mesenteric vessel, leading to massive abdominal bleeding and potential hemodynamic shock. This report presents the case of a 62-year-old patient with hypertension and anticoagulant use who arrived at the emergency department with abdominal and unusual chest pain. Initial imaging did not show signs of bleeding, delaying diagnosis until significant hemodynamic deterioration occurred, necessitating a damage control exploratory laparotomy to evacuate hemoperitoneum and ligate the middle colic artery. The double rupture phenomenon can complicate diagnosis, highlighting the importance of vigilance in patients with risk factors. CT angiography (CTA) is the preferred diagnostic method, while angioembolization is suitable for hemodynamically stable patients. Immediate resuscitation and surgical intervention are critical in unstable patients.

## Introduction

Abdominal apoplexy, also known as idiopathic spontaneous intraperitoneal hemorrhage (ISIH), is a non-traumatic large abdominal bleed due to the rupture of small mesenteric vessels. This rare but life-threatening cause of acute abdomen can result from various arterial and venous disorders. Symptoms include abdominal pain, nausea, vomiting, and hemodynamic instability. Diagnosis and treatment often face delays due to unfamiliarity with this emergency. Hence, awareness of this vascular catastrophe is crucial for prompt recognition, especially when patients present with atypical abdominal pain, gastrointestinal symptoms, cardiovascular collapse, or shock. Emergency physicians should consider this condition in patients with atypical abdominal pain and a history of cardiovascular disease [1]. We describe a case, in compliance with SCARE 2023 criteria [2], of a middle-aged female patient who presented to the emergency department with atypical chest and abdominal pain, along with uncontrolled hypertension. Initial CT indicated mild colitis; however, the patient subsequently developed a catastrophic intraperitoneal hemorrhage secondary to rupture of the middle colic artery, despite the absence of any well-defined pathology.

## Case presentation

A 62-year-old female arrived at the emergency department with sudden, sharp, and severe left upper quadrant and left chest pain, nausea, and vomiting for three hours. Her medical history included uncontrolled hypertension, chronic obstructive pulmonary disease (COPD), significant weight loss over two years, and factor V Leiden mutation managed with apixaban. She had been previously treated for hepatitis C and had a history of intravenous drug use.

**Figure 1:**
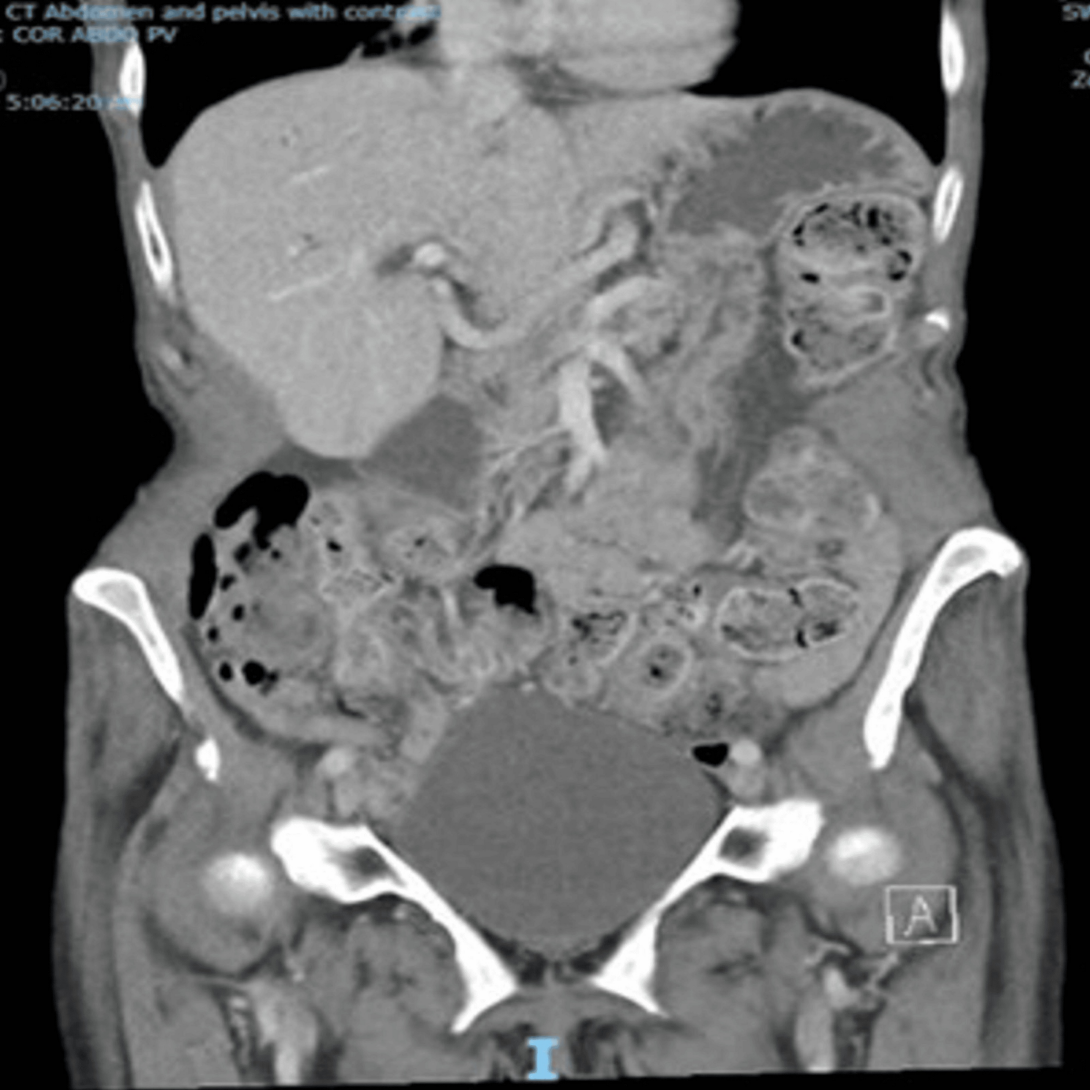
Coronal view of the initial CT revealing mild sigmoid diverticulitis, with no evidence of free fluid or aneurysm CT: computed tomography

Upon arrival at the emergency department, the patient required opioid analgesia due to pain. Her temperature was 36.5°C, her heart rate was 100 beats/min, her blood pressure was 199/110 mmHg, and her oxygen saturation was 92% on room air. She had tenderness in the lower abdomen, but no abdominal masses or peritoneal signs were detected. Laboratory tests showed a leukocyte count of 12,400/μL (reference range: 4000-11000/μL) and a hemoglobin level of 134 g/L(reference range: 116-150 g/L). Serum liver enzymes, lipase, troponin, and liver function tests were within normal limits. A contrast-enhanced abdominal CT done at 05:00 am in the morning showed mild thickening of a sigmoid colon segment with minimal adjacent fat inflammation, indicating sigmoid colitis, but no free fluid in the abdomen (Figure 1).

She received analgesia and was admitted under colorectal surgery for observation and management. She remained an inpatient with uncontrolled hypertension needing management, but required minimal analgesia.

**Figure 2:**
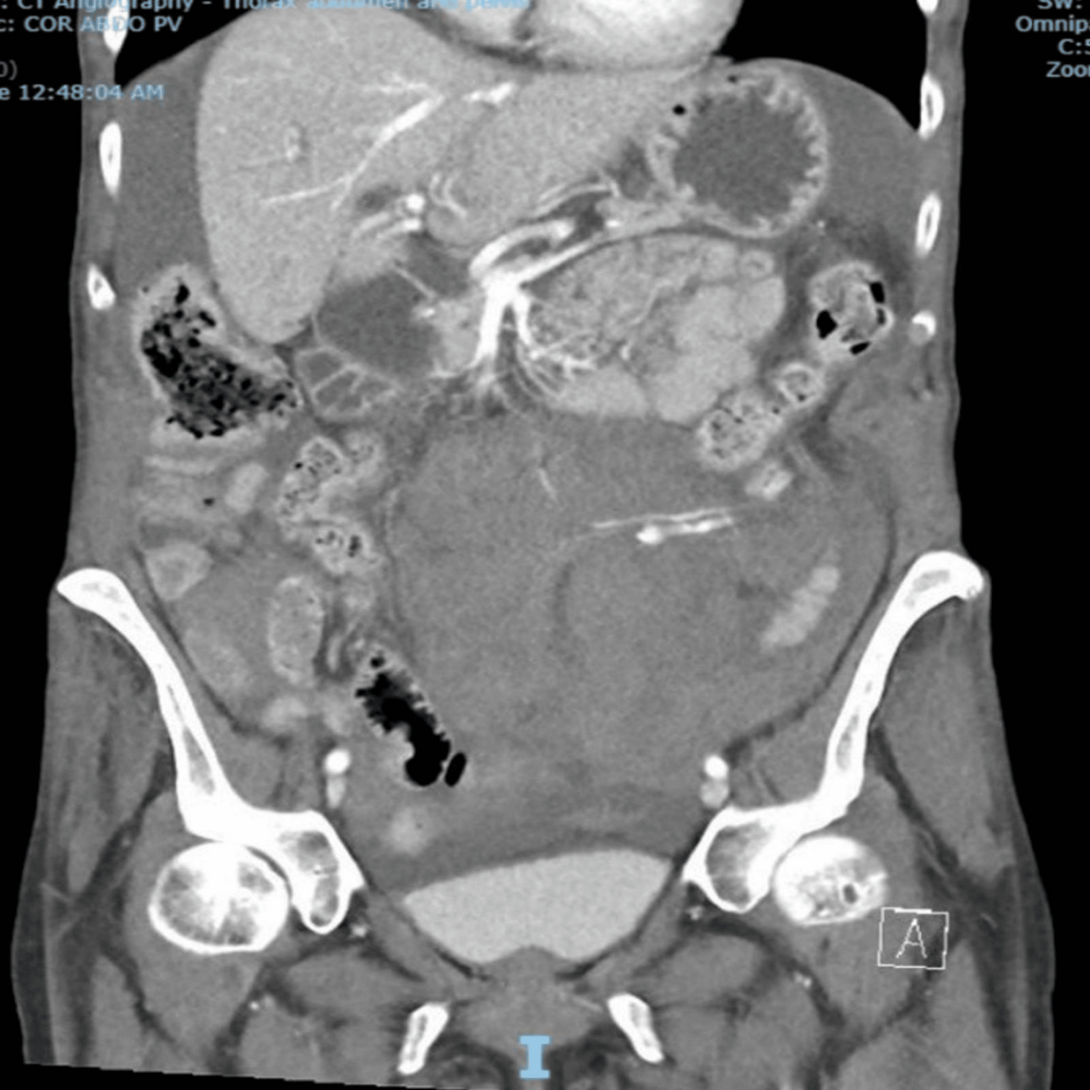
CT scan performed ~18 hours later showing large volume hemoperitoneum and hematoma with active bleeding CT: computed tomography

**Figure 3:**
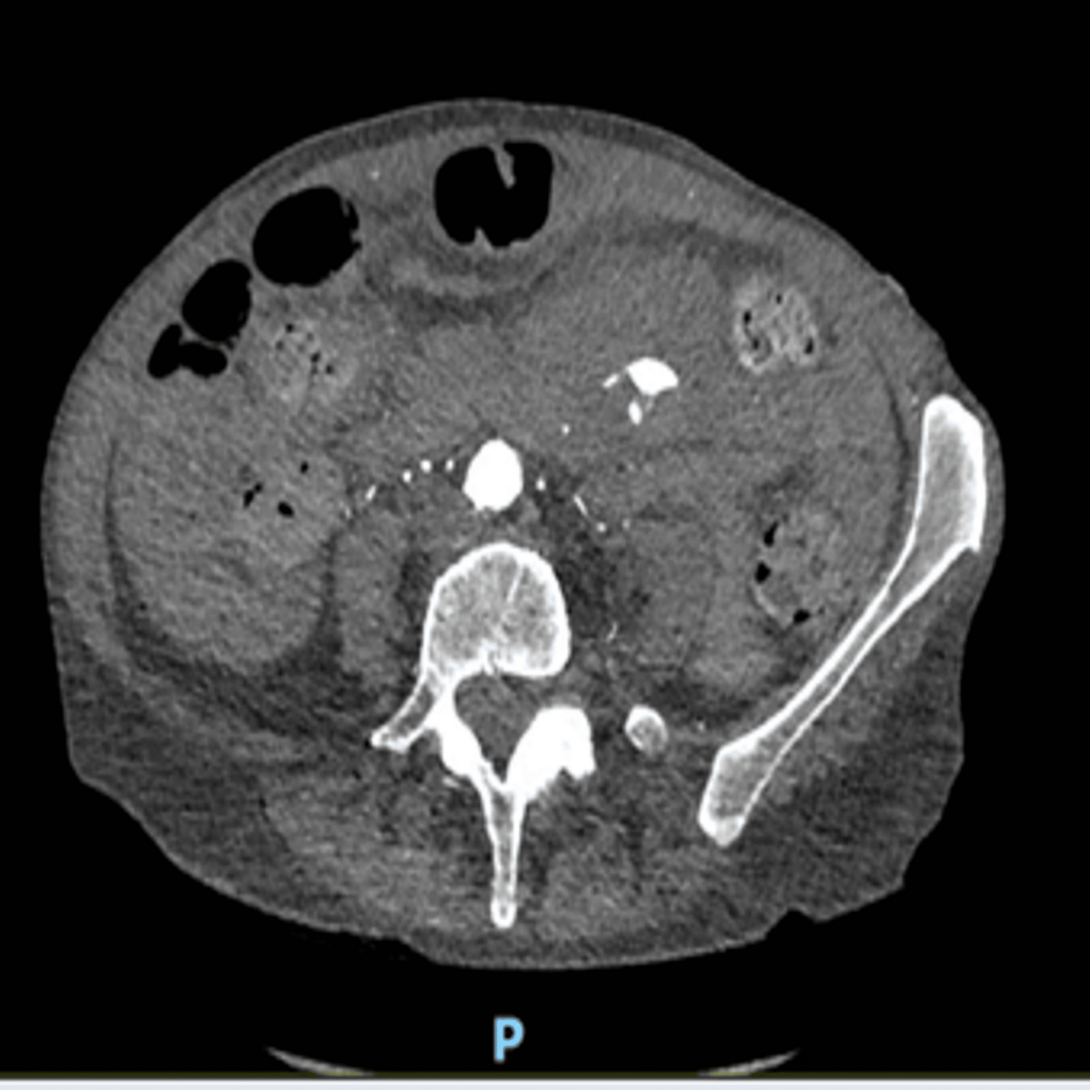
Arterial phase CT - axial view showing active blush CT: computed tomography

**Figure 4:**
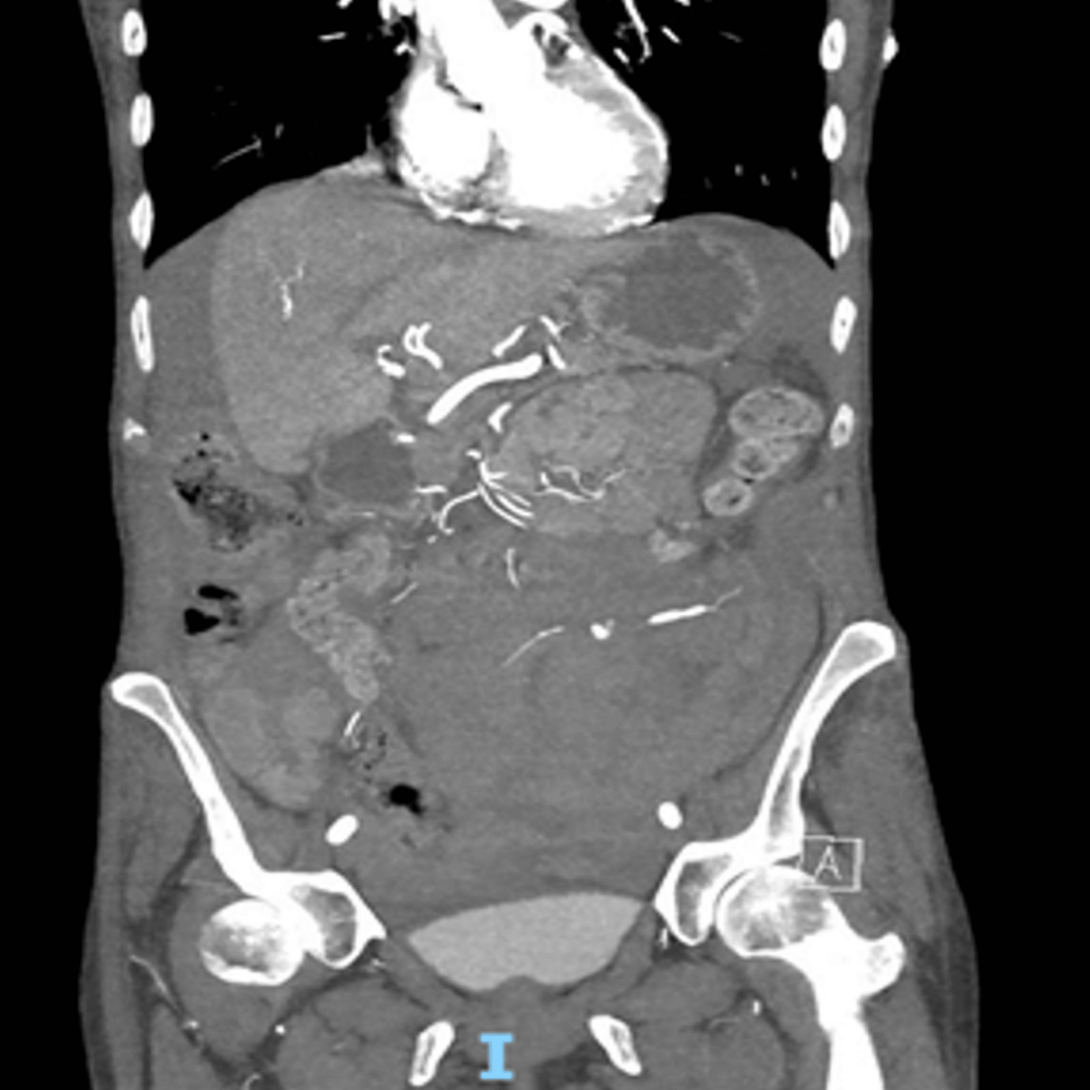
Arterial phase CT - coronal view showing active arterial blush CT: computed tomography

Approximately 18 hours post-admission, the patient's condition significantly worsened abruptly, resulting in a syncopal episode in the context of hypotensive shock. Her blood pressure decreased to a systolic measurement of 60 mmHg, and her heart rate increased to 120 bpm, accompanying the recurrence of severe generalized abdominal pain and left-sided chest pain. Venous blood gas (VBG) analysis indicated an elevated lactate level of 2.9 mmol/L (reference range: 0.5-0.7 mmol/L). Physical examination revealed a distended and diffusely tender abdomen, with signs indicative of peritonitis. Furthermore, her hemoglobin level had decreased to 70 g/L. Vasopressors were initiated, and a massive transfusion protocol was activated. A CT angiography of chest and abdomen (CTA) was done at 12:30 am, revealing a large hemoperitoneum originating from a hematoma in the left flank, with contrast extravasation indicating active hemorrhage (Figures 2-4).

**Figure 5:**
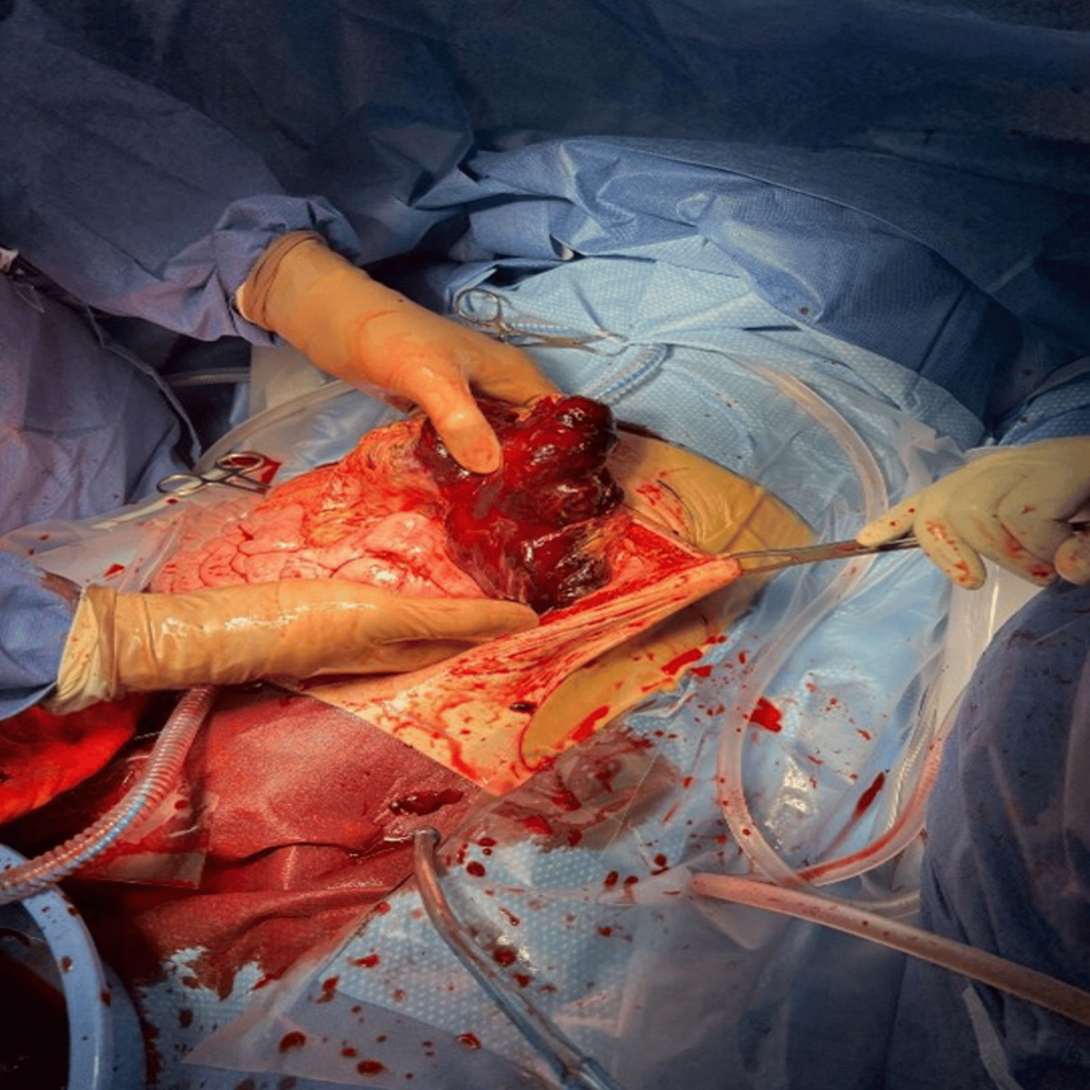
Transverse colon hematoma

The patient's condition deteriorated quickly, causing hemodynamic instability. An emergency laparotomy was required as an endovascular approach was unfeasible. An emergent laparotomy revealed a significant hemoperitoneum of 1.5 litres and a large hematoma in the transverse mesocolon (Figure 5).

A bleeding middle colic artery was identified and controlled using sutures and a LigaSure device. The transverse colon appeared healthy and viable. A laparostomy was performed following adequate hemostasis. The patient was transferred to the ICU, intubated, and sedated. Two days later, the abdominal cavity was reexamined and closed after confirming hemostasis and the viability of the colon. She was extubated the next day and discharged on the 17th postoperative day. The departmental radiology meeting reviewed her scans but found no cause of bleeding. Further investigations revealed no cardiac thrombus, autoimmune disease, rheumatoid factor, or abnormalities related to hepatitis B, hepatitis C, HIV, Treponema pallidum, or strongyloidiasis, which would suggest embolism or vasculitis.

## Discussion

The term “apoplexy” originates from the Greek apoplexia, meaning "to strike down and incapacitate." Historically, it referred to cerebrovascular stroke, known as “cerebral apoplexy”[3]. In 1931, Green and Powers named sudden intraabdominal hemorrhage as “abdominal apoplexy”[4]. The term idiopathic spontaneous intraperitoneal hemorrhage [5] is now utilized, with its definition refined to encompass peritoneal hemorrhage originating from visceral vessels, excluding instances related to established etiologies such as malignancy, trauma, iatrogenic injury, ectopic pregnancy, other gynecological conditions, or vascular diseases, including aortic aneurysm, dissection, and additional entities.

Abdominal apoplexy predominantly affects men in their fifth and sixth decades of life [5,6]. Although the precise mechanism underlying abdominal apoplexy remains unclear, it is likely related to the weakening of the tunica media in small blood vessels due to conditions such as angiopathy, aneurysms, or atherosclerosis. This vulnerability predisposes vessels to rupture, especially during sudden surges in blood pressure. Furthermore, pathology specimens frequently reveal disruption of the elastic laminae [7].

The most frequent sites of bleeding in abdominal apoplexy include the left gastric artery, the superior mesenteric artery, the splenic artery, and the middle colic artery [6-8]. About 13 cases of abdominal apoplexy caused by the short gastric artery have been reported [9]. Most ruptures occur at an aneurysm site, but 30% have no identifiable source [10]. The disparity between the locations of visceral artery aneurysms and the bleeding sites associated with abdominal apoplexy may offer valuable insights into the underlying mechanisms of this condition. It is essential to recognize that the presence of an aneurysmal stage does not necessarily precede the spontaneous rupture of a visceral artery [10]. Moreover, bleeding is often encountered in conjunction with hypertension (present in 33-50% of cases) and atherosclerosis (observed in 80-87% of cases) [6]. Consequently, alongside visceral artery aneurysms, hypertension and atherosclerosis may also play significant roles in the occurrence of abdominal apoplexy [7].

The middle colic artery (MCA) was involved in our case. It originates from the superior mesenteric artery near the pancreatic head. Aneurysms of visceral abdominal arteries are rare [11], comprising less than 3% of all splanchnic aneurysms, and are especially uncommon in the middle colic artery [12]. Out of the 44 recorded cases of MCA aneurysm, 32 involved rupture, making rupture the most common presentation [12]. The reason for the easy rupture of MCA aneurysms is unclear. However, one explanation involves segmental arterial mediolysis, causing temporal stenosis and hemodynamic changes in the visceral artery. Since the MCA connects to both the superior mesenteric artery and the inferior mesenteric artery, it may be exposed to these changes, increasing the risk of rupture, similar to pancreaticoduodenal artery aneurysms. In our patient, there were no identifiable aneurysms in the imaging performed before or during the time of the hemorrhage.

Anticoagulants, particularly warfarin, have been reported to predispose individuals to spontaneous mesenteric hematoma [13]. There are documented cases of spontaneous sigmoid mesenteric hematoma associated with rivaroxaban, where no additional pathology was identified in the surgical specimen [14, 15]. In the present case, the patient used apixaban for factor V Leiden mutation, which is a direct inhibitor of Factor Xa and could have been associated with or contributed to the massive spontaneous intraperitoneal bleed.

The clinical presentation of ISIH can vary significantly, ranging from non-specific symptoms such as dull abdominal pain and anemia to more critical conditions like acute abdomen with hypovolemic shock. According to previous case reports, a latent period of up to several hours may occur, followed by a terminal phase characterized by the rapid worsening of symptoms [5,16]. The pain is thought to be related to the speed and volume of extravasation rather than peritoneal irritation from blood. In this late phase, signs of peritonitis, hypovolemia, increasing pain, and eventually shock may become evident without prompt intervention. Our patient initially presented with severe abdominal pain, left-sided chest pain, tachycardia, and hypertension. Although her symptoms and heart rate stabilized for up to 18 hours, she subsequently experienced a sudden and rapid decline in her condition, resulting in hemorrhagic shock. This pattern of presentation may be explained by the phenomenon known as "double rupture," first described by Bockerman in 1930 in the context of ruptured splenic artery aneurysms [17].

The initial phase of hemorrhage, when contained, allows time for diagnosis and treatment. ISIH is a diagnosis of exclusion, and a CTA is a sensitive tool for assessing hemoperitoneum and helping to identify the source of bleeding. However, it seems that the early scan likely failed to identify the underlying pathology in our case.

The treatment of ISIH revolves around resuscitation and then controlling the source of the bleed. After identifying the culprit vessels by CTA, embolization can be considered if there is no suspicion for bowel compromise and if the patient is stable enough to undergo the intervention [6]. The procedure could not be performed on our patient due to significant vascular collapse. According to a literature review, most patients were diagnosed at a late stage, presenting with hemorrhagic shock. Hence, immediate surgical intervention, typically involving a lifesaving exploratory laparotomy, was necessary to identify and manage the bleeding vessel. Kleinsasser's study indicated that when the source of bleeding was located and treated, the mortality rate was 8.6%. However, if the bleeding point remained unidentified, the mortality rate could be as high as 56% [8]. Once the bleeding vessel is identified and secured, recovery is excellent with no reported recurrences. However, the condition has a 100% fatality rate without treatment.

During laparotomy, it is crucial to assess the viability of intra-abdominal organs supplied by a ruptured vessel. Our patient had a healthy transverse colon, and hence we decided against colectomy. Intra-abdominal vascular accidents often require a second-look surgery within 24-48 hours after stabilizing the patient. Our patient underwent a second-look surgery with primary closure of her abdominal wound. This approach, known as damage control surgery (DCS), significantly increases survival in critically injured patients.

## Conclusions

Abdominal apoplexy is a rare, potentially fatal vascular event that mainly affects hypertensive males. The clinical presentation of abdominal apoplexy varies, and there is no specific sign or symptom that definitively identifies it. It is important to maintain a high index of suspicion, particularly in patients presenting with a non-traumatic acute abdomen and a decrease in hemoglobin and hematocrit levels. It should also be considered in the differential diagnosis for abdominal pain in patients receiving anticoagulants. Timely diagnosis and early intervention are essential for effective management. If there is a strong clinical suspicion and previous imaging was conducted early, it is advisable to perform repeat imaging with CTA, even if the initial results were negative. Angioembolization may be considered in patients who are hemodynamically stable with low suspicion of bowel ischemia; however, most patients would need an exploratory laparotomy. DCS should be considered for unstable patients where temporary abdominal closure is necessary. In these cases, it is essential to perform a second-look surgery following DCS once the patient has been stabilized.
